# Environmental determinants of active travel in youth: A review and framework for future research

**DOI:** 10.1186/1479-5868-5-34

**Published:** 2008-06-23

**Authors:** Jenna R Panter, Andrew P Jones, Esther MF van Sluijs

**Affiliations:** 1 University of East Anglia, Norwich, UK; 2 Medical Research Council Epidemiology Unit, Cambridge, UK

## Abstract

**Background:**

Many youth fail to meet the recommended guidelines for physical activity. Walking and cycling, forms of active travel, have the potential to contribute significantly towards overall physical activity levels. Recent research examining the associations between physical activity and the environment has shown that environmental factors play a role in determining behaviour in children and adolescents. However, links between the environment and active travel have received less attention.

**Methods:**

Twenty four studies were identified which examined the associations between the environment (perceived or objectively measured) and active travel among youth aged 5–18 years. Findings were categorised according to the location of the environmental measure examined; attributes of the neighbourhood, destination and the route between home and destination.

**Results:**

Results from the reviewed studies indicated that youth active travel is positively associated with social interactions, facilities to assist active travel and urban form in the neighbourhood as well as shorter route length and road safety en-route. A conceptual framework is presented which highlights the associations between active travel behaviours and environmental factors, drawing upon both existing and hypothesised relationships.

**Conclusion:**

We provide a review of the available literature and present a novel theoretical framework that integrates the environment into the wider decision making process around travel choices for children and adolescents. Further work should explore associations where gaps in understanding have been identified, and account for the main moderators of behaviour so hypothesised associations can be confirmed.

## Background

Physical inactivity is a risk factor in the development of a range of diseases, such as coronary heart disease and type 2 diabetes [1]. Engagement in physical activity is vital for the prevention of obesity [2], osteoporosis [3], and cardiovascular disease [4]. It has also been associated with positive effects on mental health [3]. In the UK, levels of physical activity amongst children are low. Recent surveys report that 3 out of 10 boys and 4 out of 10 girls fail to meet recommendations [5]. This is despite the fact that being sufficiently active can be achieved by regularly engaging in moderate intensity exercise such as walking or cycling; activities that can be incorporated into everyday life for recreation or transportation. These types of exercise have positive health benefits, irrespective of the purpose [6].

Walking or cycling for transport, otherwise known as 'active travel', is one way in which children can increase their levels of physical activity. Walking is popular, convenient and free and has even been described as a "near perfect exercise" [7]. Although travel by bicycle does introduce health risks through accidents and injuries [8], the health benefits of cycling have been shown to outweigh these risks [9]. In spite of their health benefits and the variation in the method of assessment used [10], the number of walking and cycling trips undertaken by children is low. In the United States of America (US), only 10% of children walk to school [11] whereas in Scandinavian countries the prevalence of active travel is much higher [12]. In addition, in the United Kingdom (UK) [13], US [14] and Australia [15] there is evidence that the number of children walking to school is decreasing.

A number of studies have examined the contribution of active travel to overall activity levels. They have generally found that children who walk to school are likely to engage in more physical activity overall [16] and are more likely to meet physical activity guidelines [17] than children who travel by motorised travel. For example, Cooper et al. [18] showed that boys who walked to school were more active after school and into the evening than those who travelled by car.

Understanding the characteristics of children who walk or cycle, and the reasons for choosing these travel modes, are important first steps in developing effective interventions to increase the number of children engaging in active travel. Interventions that modify environments to make them more amenable for walking and cycling may be particularly attractive as they provide the potential for sustained impacts on whole populations [19], especially if accompanied by other determinants such as parental support, friend support and self-efficacy [20].

In recent years, there has been a significant growth in the number of studies that have examined the association between active travel and the environment in adults [21]. Environmental factors such as connectivity, urban form, and the provision of sidewalks and cycle paths have been shown to be associated with walking and cycling for transport [22]. However, the influential factors may be different for children. For a younger age group, travel choices may be more strongly influenced by traffic safety concerns and the views of parents, for example, and this may mean that the determinants are rather different to those observed in adults.

Relatively little is known about the relationship between environmental factors and children's active travel behaviours. In fact, a recent editorial highlighted the need for greater research into the social and environmental determinants [23]. We argue that a key reason why current research in children is limited is the absence of a comprehensive theoretical framework that explains how the environment may influence active travel. McMillan [24] has developed a framework relating urban form with travel mode choice for a trip to school. It identifies the key decision maker as the parent, and highlights the mediating and moderating factors which influence their decisions. Whilst a useful contribution to the field, the framework fails to incorporate the varied components of the environment which have been examined in the literature which may influence parental decision making. In addition, it is not necessarily applicable to other types of travel behaviours in which children may engage, such as travel to a friend's house, parks or local destinations. These important yet informal types of activity have often been overlooked in physical activity research [25]. A second framework, developed by Pikora et al. [26], also identifies those specific components of the environment which influence walking and cycling for both leisure and transport. It is based on published evidence, policy literature and interviews with experts. Elements of the environment are divided into four categories; safety, functional, aesthetics and destinations. This framework highlights the importance of attributes of the residential neighbourhood and destinations within the neighbourhood that are within walking or cycling distance. However, for populations to engage in active travel behaviours, it is also likely that attributes of a route between these two locations will be important. Furthermore, the framework is not specific to children, whose travel needs and their associated influences might be different to adults.

This paper critically reviews the existing literature on the environmental influences on active travel behaviour in children and, using this evidence-base, updates previous work by presenting a new comprehensive framework within which the environmental determinants of children's travel behaviour may be studied.

## Methods

Quantitative studies examining the association between environmental attributes and active travel behaviours were identified using computer database searches of PyschInfo, PubMed and Medline. Search terms included walking, cycling, transport, physical activity, active commuting, neighbourhood, and school. To limit the search to the population of interest the terms child, children, adolescent and adolescence were also included. The reference lists of identified studies were also reviewed for additional studies. Studies were included if they 1) examined walking or cycling as a mode of transport as an outcome variable 2) included at least one environmental dependent variable and 3) had a sample of youth between the ages of 5–18. All studies meeting these criteria were included regardless of whether they used self reported or objectively recorded measures of environmental characteristics or travel modes. Studies which used motorised travel as an outcome, for example those examining the determinants of being driven to school [27], were not included in the review. Studies were classified as examining children if the majority of the sample were between the ages of 5 and 11. Adolescents were defined as individuals between 12 and 18 years of age. This definition has been used in a previous review [28]. Where ages are not differentiated within this range or where the sample spanned both age groups the term 'youth' is used throughout this review.

## Results

### Studies identified

A total of twenty-four studies were identified as providing evidence for the framework development. They came from a variety of different fields including health promotion and physical activity [25], transportation [29] and planning [24]. Most research focussed on walking and cycling to school (n = 19), with only two studies examining other local destinations. The majority of studies reviewed here were conducted in the US (13) and Australasia (7), with only four studies from Europe. Only one study [30] used an objective method of assessing travel mode; student observation. The remaining studies used self-reported measures of active travel behaviour. Of these, 10 used self-reported travel mode from the parent, 8 from the child and 5 from travel diaries. Environmental variables were measured using objective methods of assessment (11 studies), self-report methods (10 studies), and combinations of objective and self-report methods (3 studies). Of the 10 studies which included only self-reported assessment of the environment, 4 used parental report, 3 used child report only, whilst 3 used both parent and child report of the environment. Table 1 gives a summary of the characteristics and main findings of the studies reviewed.

**Table 1:** Characteristics and main findings of the studies reviewed

**First Author Date**	**Number/Gender/Country**	**Age group (years)**	**Design**	**Environmental attributes (independent variable)**	**Active travel behaviour (outcome variable)**	**Significant associations (p < 0.05) with outcome variable**
Alton 2007 [33]	473 M/F UK	9–11	CS, P	Child perceptions of traffic, road safety, strangers, provision of recreational facilities, parental concerns about traffic and safety.	Child self-reported walking trips in the last week.	More walking associated with heavy traffic and unsafe streets.
Boarnet 2005 [54]	1244 M/F US	3^rd^–5^th ^grade (˜8–11)	I, O	Presence of sidewalks, crossings and traffic control.	Parent reports of walking or cycling to school.	Those passing new sidewalks and traffic controls more likely to show increases in walking.
Braza 2004 [47]	2993 M/F US	5^th ^grade (˜9–11)	CS, O	School size, population density and number of intersections per street mile around school.	Child self-report of walking and biking to school on one day.	Smaller school size and higher population density around school associated with higher levels of walking.
Bruijin 2005 [48]	3859 M/F Nether-lands	High school (˜12–18)	CS, O	Objectively assessed level of urbanisation of residence.	Adolescents self-reported use of a bike for transport.	Those living in less urbanised places more likely to report cycling for transport.
Carver 2005 [25]	347 M/F Australia	12–13	CS, P	Parent perceptions of recreational facilities, general safety, traffic, and good places to be active. Adolescent perceptions of ease of transport by bike, personal safety, traffic safety, strangers, social interactions, unattended dogs, strangers and provision of retail food facilities.	Adolescents self-reported frequency of walking to school and for transport Adolescents self-reported frequency of cycling to school and for transport.	Walked or cycled when good sports facilities (M), social interactions in the neighbourhood (MF), roads safe (MF), and convenience stores further from home (F).
Cole 2007 [49]	559 M/F Australia	4–7	CS, P	Parental report of distance to school.	Parent report of no. of days walking/cycling to school over last 5 school days.	Those living further from school less likely to walk or cycle to school.
Evenson 2006 [36]	480 F US	10–15	CS, P	Adolescent perceptions of personal & traffic safety, high crime, seeing others playing, unattended dogs, well lit streets, many places within easy walking distance of home, ease of walking to bus stop, presence of trees, exhaust fumes and bicycle or walking trails.	Adolescent self-report of no. of days walked or cycled to school in past week.	Less likely to walk or cycle to school if no exhaust fumes/bad smells in the neighbourhood (F). More likely if bicycle or walking trails and facilities were present (F).
Ewing 2004 [42]	726 U US	5–18	CS, O	Objective assessment of sidewalk width, proportion of street miles with trees, bike lanes, sidewalks, estimated walk/bike time between destinations, school size, population and employment density.	Travel diary of mode of travel to school.	Those with shorter walk or bike times to school and routes with sidewalks on main roads more likely to walk or cycle to school.
Frank 2007 [45]	3161 M/F US	5–20	CS, O	Intersection density, residential density, mixed land use, at least 1 commercial land use and at least 1 recreation/open space land use.	Self-reported travel mode from two day travel diary.	Recreation space associated with more walking. All environmental variables associated with more walking in 12–15 year olds. Higher residential density associated more walking in 9–11 year olds. At least 1 commercial land use & higher intersection density associated with more walking in 16–20 year olds.
Fulton 2003 [43]	1395 M/F US	4^th^–12^th ^grade (˜8–18)	CS, P, O	Parent reported urban/rural status. Youth perceptions of neighbourhood safety and presence of sidewalks.	Youth self-report of normal mode of travel to school.	Living in an urban area and having sidewalks in the neighbourhood associated with more walking.
Hohepa 2007 [41]	3471 M/F New Zealand	12–18	CS, P	Adolescent perception of social support from parents, siblings, and school	Adolescent self-report of no. of trips walking/cycling to school over last 5 school days.	Amongst 12–16 year olds, social support from friends & school associated with more walking or cycling to school. Amongst 16–18 year olds, no associations found.
Kerr 2006 [35]	259 M/F US	5–18	CS, P, O	Parent perceptions of residential density, land use mix, stores within 20 mins walk, street connectivity, walking or cycling facilities, crime, pedestrian safety, aesthetics and parental concerns. Objectively assessed intersection density, residential density, land use mix, neighbourhood & individual walkability.	Self-reported travel mode to school from two day travel diary.	More active commuting associated with higher land use mix, more stores within 20 mins, greater street connectivity, more walk and bike facilities, more aesthetically pleasing neighbourhood, fewer parental concerns, higher residential density, individual & neighbourhood walkability.
Kerr 2007 [46]	3161 M/F US	5–18	CS, O	Objectively assessed neighbourhood intersection density, residential density, mixed land use, ≥ 1 commercial land use and ≥ 1 recreational land use.	Parental report of travel mode to school.	More walking for transport with greater intersection and residential density, mixed land use, ≥ 1 commercial land use, ≥ 1 recreational land use. In non-whites, more walking with mixed land use and ≥ 1 recreational facility. In whites, all measures associated with walking.
McDonald 2007 [29]	614 M/F US	5–18	CS, O	Objectively assessed distance to school, dwelling units per sq km, land use mix and average block size.	Self-reported travel mode to school from two day travel diary.	Those with journey length of <1.6 km more likely to walk to school and smaller block size associated with more walking/cycling. For longer trips, higher dwelling units per sq km associated with more walking/cycling.
McMillan 2007 [38]	1128 U US	3^rd^–5^th ^grade (˜8–11)	CS, P O	Parent perception of neighbourhood safety and traffic speeds > 30 mph on route to school. Objective measurement of proportion of street segments with a complete sidewalk system, >50% of windows facing the street and a mix of land uses.	Parental report of travel mode to school.	More likely to walk or cycle to school when distance to school < 1 mile, neighbourhood had mixed land use & greater amount of windows faced street. Less likely when traffic speeds > 30 mph and unsafe neighbourhood reported.
Merom 2006 [50]	808 M/F Australia	5–12	CS, P	Parental perception of distance to school and road safety.	Parental report of travel mode to school during a usual week.	Those further from school and having unsafe the neighbourhood less likely to walk or cycle to school.
Mota 2007 [32]	705 F Portugal	7^th^–12^th ^grade (˜11–18)	CS, P	Parent perception of access to destinations, street connectivity, facilities for walking and cycling, safety, social environment, aesthetics and provision of recreational facilities.	Parental report of travel mode to school.	More likely to walk to school when streets in the neighbourhood were more connected.
Schlossberg 2005 [52]	104 U US	Middle school (˜11–14)	CS, O	Objectively assessed network and straight line distance to school	Parental report of walking or cycling to school frequency.	More likely to actively commute if distance to school is shorter using both measures. However, no statistical significance is given.
Schlossberg 2006 [51]	287 M/F US	6^th^–8^th ^grade (˜11–14)	CS, O	Objectively assessed distance to school, intersection density and dead end density of route, route directness, major roads and rail-roads proximal to route.	Parental report of walking or cycling to school frequency.	Shorter distance to school associated with more walking and cycling. Higher intersection density and lower dead-end density associated with more walking.
Sirard 2005 [30]	U U US	Elementary school (˜6–12)	CS, O	Objectively assessed school SES and level of urbanisation around school	Direct observation of prevalence of walking or cycling to & from school.	No significant associations identified.
Sjolie and Thuen, 2002 [53]	88 M/F Norway	14–16	CS, O	Objectively assessed urban rural residence and distance to school.	Adolescent reports of number of times walked or cycled to activities in a week.	Those in an urban area and having shorter distance to travel likely to report more walking or cycling to school & for transport.
Timperio 2004 [34]	1210 M/F Australia	5–6 and 10–12.	CS, P	Parent perceptions of heavy traffic, safety (road, strangers), no lights or crossings, need to cross several roads to reach play areas, limited public transport & not many other children around. Child perceptions of traffic, safety (road, strangers) and provision of parks or sports grounds.	Parental report of number of times walking or cycling used to get to destinations.	For those aged 5–6, less walking or cycling associated with heavy traffic (M) & limited public transport (F). For those aged 10–12, less walking or cycling associated with no lights or crossings (M), need to cross several roads to reach play areas (MF), limited public transport (F), & few parks and sports grounds near home (F).
Timperio 2006 [40]*	912 M/F Australia	5–6 and 10–12.	CS, P, O	Child and parent perceptions of heavy traffic, strong concern about strangers and road safety, no lights/crossings, need to cross several roads to reach play areas, limited public transport & not many other children around. Objectively assessed distance to school, busy road barrier, route along busy road and pedestrian route directness.	Parental report of walking or cycling to school frequency.	Less likely to walk or cycle to school if journey to school > 800 m and busy road en-route. In those aged 5–6, a steep incline en-route associated with less walking or cycling. For those aged 10–12, a direct route associated with less walking or cycling.
Ziviani et al. 2004 [39]	164 M/F Australia	1^st^–7^th ^grade (˜6–11)	CS, P	Parent perceptions of distance to school, traffic, manned crossings and pollution in the neighbourhood	Parental report of walking or cycling to school at least once a week.	Those with shorter journeys to school and whose parents had no concerns about road hazards or personal safety, more likely to walk to school.

Note:

Number/Gender/Country: M; male, F; female U; unknown.

Design: CS, cross-sectional; I, intervention; P, perceived environment; O, objectively measured environment.

*Given that the same sample was used in Timperio (2004) and Timperio (2006), only new findings from the 2006 have been included under the 2006 study.

The environmental variables examined fell broadly into three categories; the attributes of the residential neighbourhood, the destination, and the routes between home and destination. This evidence review is structured accordingly. Table 2 presents these findings according to the age of sample (youth, children, and adolescents) and the environmental characteristics examined.

**Table 2:** Summary of associations between physical environmental characteristics and active travel behaviour

	**Associations with active travel behaviour**								
Sample Age	**Youth**			**Children**			**Adolescents**		
Direction of Association	Negative	None	Positive	Negative	None	Positive	Negative	None	Positive

**Characteristics of the neighbourhood**									
Provision of facilities			[35]		[33,34]		[25]F	[32,34]M [36]F	[25]M [34]F
Personal safety			[35]		[33,34,40]	[38,39]		[25,36]F	
Road safety				[34] M	[34]F	[33,34,40]		[36]F	[25]
Social interactions			[29]					[36]F	[25,40,41]
Facilities to assist active travel			[35,42,43]					[32,36]	
Urban form and street design			[29]		[45]	[32,35]			[45]
Aesthetics			[35]				[36]		

**Characteristics of the destination and surroundings**									
*Destination characteristics*									
School size		[42]		[47]					
*Characteristics of surroundings*									
Urban-rural status	[48]	[30]							
Facilities assist active travel					[38]				
Urban form						[38]			
Visibility						[38]			

**Characteristics of the route**									
Length	[52]			[40,49]			[51,53]		
Road safety			[54]			[38,40,50]			
Urban form & topography				[40]	[40]		[40]	[51]	[51]

Numbers given are reference numbers.

Effects which are specific to different gender groups are noted separately; M male; F females.

The same study may occur twice within a topic if different measures are used and show different associations.

### Components of the identified characteristics

#### 1) Characteristics of the neighbourhood environment

The neighbourhood environment within which a child lives is likely to be particularly important in determining their decision about travel modes, because a child and their parents come into daily contact with it. Hence it has commanded the most attention and provides the largest volume of research.

#### Provision of facilities

Environments which support walking for travel purposes tend to provide shorter distances to frequently travelled locations such as commercial areas, bus stops and recreational locations. In these 'more walkable' areas residents tend report higher numbers of walking or non-motorised trips [31].

Two Australian studies, one Portuguese study and one other study undertaken in the UK examined the perceived provision of recreational or sporting areas and active travel. Of these, one study reported parental perceptions only [32] and three examined both parental and youth perceptions [25,33,34]. In adolescents, Carver et al. [25] found that boys whose parents reported that their neighbourhood had good sports facilities tended to report more cycling for transport. This association was not evident in girls, or in walking behaviours for either gender. In contrast, Alton et al. [33] found no association, between parks or sports facilities in the neighbourhood and walking when children were asked, after adjustment for confounding factors, such as age, sex and ethnicity. Similarly, in older children, Timperio et al. [34] found no evidence that trips were more common in areas where parents reported more recreational facilities. However, when children were questioned, girls, although not boys, who reported having no parks near where they lived were less likely to walk or cycle for transport.

The presence of destinations or shops in close proximity to a youth's home has also shown mixed associations with active travel, varying according to gender and whether parental or child perceptions were examined. Evidence examining parental perceptions of distance in Seattle, USA suggested that youths whose parents reported having stores within a 20 minute walk of their home were 3.2 times more likely to report walking or cycling to school [35]. However, Mota et al. [32] found no association between parental reports of destination accessibility and active commuting in adolescents. Indeed, findings of work considering adolescents' own perceptions have generally been equivocal. Evenson et al. [36] identified positive associations between girls own knowledge of the number of destinations in the neighbourhood and walking or cycling to school, but having many places they liked to go in their neighbourhood was not associated with active travel behaviour. In a study of Australian adolescents, girls who reported having convenience stores near to home were actually less likely to walk for transport at the weekends, with no association observed during the week [25].

#### Safety

Parental concern about safety is often cited as a barrier to walking and cycling. Safety is a complex concept as it includes many components. Studies that have examined parental fears for their children's safety suggest that the main components are personal and road safety [37]. This section addresses these two aspects of safety.

##### i) Personal safety

Research examining parental or youth concerns about personal safety have produced mixed associations. Eight studies examined the associations between active travel and parental concerns about safety, with three [35,38,39] reporting that greater parental concerns were associated with youth being less likely to regularly walk or cycle to school. The studies examined concerns about neighbourhood safety in general [25,35,39], or safety whilst walking alone in children [38] and in adolescents [36]. The strongest association was reported by Kerr et al[35] who found that youth whose parents who had lower general concerns about their safety, either on their route or in their neighbourhood, were 5.2 times more likely to walk or cycle to school. However, in children no association was found between child or parental concern about strangers and walking or cycling [40].

Four other studies, undertaken in Australia, [25,34], the UK [33], and the US [36] found that neither parental [34], child [33] or adolescent concerns [25,36] about personal safety were associated with walking and cycling to local destinations. Timperio et al. [34] suggested that this lack of association may be unsurprising given the high prevalence (over 80%) of concern about strangers.

##### ii) Road safety

Five studies examined the association between road safety and active travel. One investigated associations with active commuting to school [40] and four with active travel in the neighbourhood [25,33,34,36]. Timperio et al. [40] found that children whose parents reported that there were no lights or crossings in the neighbourhood, and who had to cross busy roads to get to school, were less likely to actively travel to school. Carver et al. [25] found that in adolescent girls, perceptions of safe roads in the neighbourhood were positively associated with walking to destinations. Similarly, adolescent boys whose parents reported that that traffic made it difficult or unpleasant to walk in their neighbourhood were less likely to report walking or cycling in the neighbourhood. Alton et al. [33] also found that unsafe roads were associated with a lower prevalence of walking in children, regardless of whether the child or parent reported safety. A study by Timperio et al. [34] using parental perceptions noted different findings according to child age and gender. Older boys whose parents perceived that there were no lights or crossings for their child to use were less likely to report walking and cycling in the neighbourhood. But no such associations were noted in girls or younger children. Nevertheless, boys aged 5–6 whose parents reported heavy traffic in the neighbourhood were more than twice as likely as others to walk or cycle to destinations at least three times a week, whilst older girls whose parents reported a need to cross several busy roads to reach play areas were less likely to walk or cycle.

##### Social interactions

Five studies have reported positive associations between social interactions and active travel in children [25,29,36,40,41]. In adolescents, low peer support was associated with a reduced odds of active travel [41]. Carver et al. [25] found that adolescents, particularly girls, who had friends living nearby, young people the same age to socialise with, and knew and waved or talked to their neighbours were more likely to report walking and cycling in the neighbourhood. For boys, having lots of children the same age to socialise with was also associated with more cycling for transport, but not for any other active travel behaviours. In addition, McDonald [29] investigated the influence of social cohesion on youths' travel patterns in California. She found that when trips were stratified by length, measures of social cohesion were only promoters of trips shorter than 1.6 km.

In the USA, Evenson et al. [36] found no association between girls reporting that they often saw other children playing outdoors and active travel behaviours. However, two studies [25,40] both found that children whose parents perceived few other children in the neighbourhood for their child to play with were less likely to actively travel to school, possibly as there were fewer opportunities to walk to school in the company of others.

##### Facilities to assist active travel

It may be expected that the presence of facilities such as sidewalks and cycle paths would encourage walking and cycling. However, five studies [32,35,36,42,43], all except one conducted in the USA, have produced mixed results.

In a large study of elementary school students aged 5–18 in Florida, Ewing et al. [42] found that students were more likely to walk to school if there was higher sidewalk coverage around their school and home. Two further studies, which examined parental report of sidewalks [43] or sidewalks and cycle paths [35], found that the presence of these features were associated with increased levels of active travel. Indeed, Fulton et al. [43] found that youth whose parents reported having sidewalks on most of the streets in their neighbourhood were over 4 times more likely to report normally walking or cycling to school. Despite this, Evenson et al. [36] found no association between active commuting and adolescent girls' own perceptions of a presence of sidewalks on most streets in the neighbourhood, and neither did Mota et al. in a sample of Portuguese adolescents [32], although, Evenson et al. [36] did find that girls were more likely to walk or cycle to school if bicycle or walking trails were present.

##### Urban form and street design

The term 'urban form' relates to a number of measures which capture the structure and connectivity of an urban area [22]. Measures of urban form often include elements such as residential density or land use mix. Other indicators include connectivity (for example how easy it is to walk between two points in the neighbourhood using sidewalks), the accessibility of facilities, and dead-end or cul-de-sac density [44].

Five studies highlight positive associations between urban form and active travel behaviours in children [29,32,35,45,46]. Three examined self-reported [29,32,35] and two objective [45,46] measures of the environment. McDonald [29] found that the effects of the built environment on travel behaviour may differ according to the trip length. In that analysis, mixed land use and a greater number of dwelling units were associated with the use of active travel modes for longer, but not shorter trips. Frank et al. [45] suggested that the effects of urban form may vary according to the age of children. For adolescents, higher residential density, a mixed land use, having at least one commercial land use and at least one recreational space in the neighbourhood were associated with walking for transport. Proximity to recreational land uses was the only dominant correlate of walking for transport in children [45]. Self-reported land use mix and street connectivity of the neighbourhood showed positive associations with children's active travel behaviour in two studies [32,35]. In one of these, Kerr et al[35] found neighbourhood residential density had the strongest association with active travel to school, with youth in the top tertile of density being 3.2 times more likely to walk or cycle compared to those living in lower density areas.

##### General aesthetics

Only two studies [35,36] have examined the association between neighbourhood aesthetics and active travel. Both were undertaken in the USA and produced contrasting findings. Evenson et al. [36] found that adolescent girls who reported exhaust fumes or other bad smells in their neighbourhood were more likely to report active travel, most likely because these active travellers would be more exposed to those environmental problems. The presence of trees, interesting features to look at or a lack of litter were not associated with active travel. However, Kerr et al. [35] report that those youth whose parents believed their neighbourhood was aesthetically pleasing were 2.5 times more likely to report active commuting compared to those rating their neighbourhood as less pleasing.

##### 2) Characteristics of the destinations and their surrounding environment

Few studies have examined the association between travel behaviour and attributes of the area around destinations or of the destinations themselves. For example, the presence of a busy road in close proximity to a destination may deter children from walking or cycling to it even if their residential neighbourhood is traffic free.

Only five studies have examined the association between the physical environment around schools and travel behaviours in children. Most used objective methods of assessing the environment, including street section audits [38] and computer mapping [47].

Sirard et al. [30] found no association between walking or cycling to school and levels of urbanisation around four elementary schools in urban and suburban locations. In contrast, de Bruijin et al. [48] found that Dutch adolescents attending schools in less urbanised cities (those with less than 50,000 inhabitants) were more likely to use their bicycle for transport than those living in more urbanised areas. Braza et al. [47] examined the association between rates of walking and cycling and school neighbourhood design at thirty-four elementary schools. Small school size and a high local population density were associated with an increased likelihood of active commuting in children, although Ewing et al. [42] found school size not be an important factor in determining walking and cycling in youth. McMillan [38] studied the micro-level characteristics of urban form surrounding the school, concluding that in the areas where windows of buildings faced the streets and where mixed land uses were present, children are more likely to report active travel. However, the presence of sidewalks on both sides of the street around the school was not associated with active travel.

##### 3) Characteristics of the routes between destinations and home

Rather little work has been undertaken examining the association between the characteristics of children's travel routes and their travel behaviour. Amongst the studies that have been published, the route to school is most frequently examined, with both subjective and objective methods of route attribute quantification being used.

##### Length of route

Unsurprisingly, length of route to school was found to be a significant predictor of travel behaviour in all studies, with those who had shorter journey distances being more likely to walk or cycle to school [40,49-53]. For example, Timperio et al. [40] examined the influence of route length for children aged 5–6 years and 10–12 years separately. Distance to school was more important in determining active travel behaviour in the older children, a likely result of them seeking independence from their parents. For both groups, those who had a journey to school of less than 800 m were over 5 times more likely to report walking or cycling to school than those whose journey was greater. Yet for children aged 10–12 years, those living most proximal were over 10 times as likely to walk or cycle. Unsurprisingly, the strength of association between active travel and distance appears to vary with travel mode; Schlossberg et al. [51] noted that the effect of distance is greater for walking than cycling.

##### Road safety on the route

Three studies have investigated the associations between active travel and traffic safety en-route to school [38,40,50]. Each found a measure of traffic safety to be associated with higher levels of walking or cycling for transport. The measures were the presence of roads en-route where the speeds of vehicles were slow [38] which were not busy [40], and routes where parents perceived the road was safe [50].

Boarnet et al. [54] undertook an evaluation of the Safe Routes to School (SRS) programme in California. The programme provides funding to improve the environment for active travel to and from school. Changes included sidewalk and crossing improvements and traffic controls [54]. The authors reported that, after the programme implementation, children who passed environmental improvements were more likely to show increases in active travel to school than children who did not pass projects on their route.

##### Urban form & topography

Two studies have investigated the associations between active travel and measures of urban form en-route to school, including connectivity and intersection density [40,51]. Both used computer mapping to calculate routes and identify features, but they report mixed findings.

Timperio et al. [40] found that adolescents who had a more direct route to school were actually less likely to report walking or cycling, suggesting a disconnected environment may represent a safer one for walking or cycling as a mode of transport. This finding contrasts with those reported by Schlossberg et al. [51] who found no association with route directness but that children whose routes had higher intersection and lower dead end densities were more likely to walk, but not cycle, to school.

One study examined active travel and the topography of the urban environment. A steep incline on the route to school was associated with a lower prevalence of walking and cycling for children aged 5–6, but not those aged 10–12 years [40].

### A conceptual framework for youth's active travel

**Figure 1:**
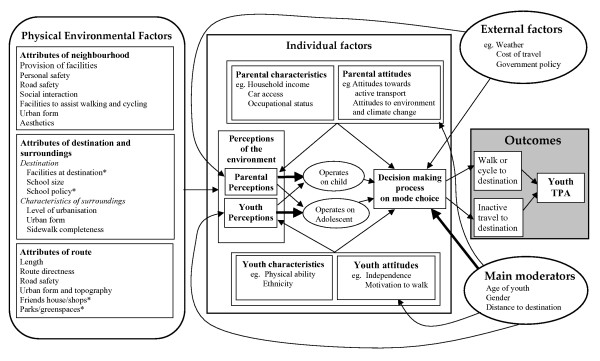
**A conceptual framework for the environmental determinants of active travel in children**. * Not studied in relation to active travel behaviour in children. TPA = Transport-related Physical Activity. Arrows indicate a hypothesised direct relationship. Larger thicker lines indicate a stronger hypothesised direct relationship.

Based on the evidence presented in this review, a new conceptual framework has been created and presented in Figure 1. This framework builds on previous work in two ways. Firstly, it highlights two main moderators of behaviour which alter the strength of the association between the physical environment and active travel in children; age of youth, gender and distance travelled. Secondly, from this review, it is evident that a broad range of environmental characteristics have been examined in relation to children's active travel, whilst McMillan [24] uses just urban form as a core element of her framework. Hence, in this new framework we have encompassed diverse physical environmental factors including characteristics of the neighbourhood, destination and route environment. These have all been associated with active travel behaviours in youth and we therefore suggest that a broader view which considers a wider range of factors is appropriate.

The framework contains four main domains of influence on active travel behaviour: individual factors, those associated with the physical environmental, external factors outside the most proximal domains of influence, and main moderators. We suggest that the individual, physical environmental and external domains are most likely to influence decision making regarding mode of travel, while the main moderating factors will alter the strength and form of the association between those factors and the decision made. McMillan [24] suggests that in children up to a certain age, parents are the main decision makers about mode of travel. In this framework, we accommodate both children and adolescents. Nevertheless, the framework recognises that either parents or youths may decide how to travel, with the main outcome being the level of transport related activity. In those who travel by car, this will be relatively low and in those who walk or cycle for whole or part of the journey the level of activity will be higher.

It is likely that all three types of physical environmental factors grouped in Figure 1 will have an influence on both parental and youth perceptions of the suitability of the environment for active travel. Yet these perceptions may be formed as a result of the actual attributes of the physical environment, or based on pre-existing opinions or views. Our framework allows for the fact that the actual decision on travel mode is likely to be a result of both parental and child perceptions. Evidence from the retail sector consistently indicates that parents are influenced by children's opinions when making purchasing decisions [55,56]. We believe that similar processes will operate with regard to children's travel mode choice, and that most children and their parents will enter into a dialogue during the decision making process. From this review, it is evident that parental perceptions of environmental characteristics are generally associated with children's behaviour, yet children's own perceptions are less consistently associated with their own behaviour. For adolescents, the influence of parental perceptions of the environment may be less important, yet further research should explore the influence that parents, children, and adolescents have in the travel mode choice process.

In the framework, those physical environmental factors for which research evidence does not exist, but which we believe are likely to be associated with active travel, are marked with an asterisk (*). These include the role of the provision of facilities at the destination. For example, having well-maintained, and covered cycle storage in schools may encourage active travel, yet the influence of such physical facilities, and associated school policies leading to their provision, has not been investigated. Children may also be more likely to walk or cycle to destinations if there are parks to play in en-route, or shops or friends' houses to visit. Future research should examine these characteristics in more detail.

Youth characteristics and attitudes will clearly influence their decision to walk or cycle, and the key ones are identified in the framework. Those youths who are motivated to use active travel modes because of perceived independence and freedom from parents are more likely to walk or cycle [57] or influence their parents' decision about travel mode. It is also hypothesised that attitudes may influence perception of the environment. Those with positive attitudes such as feeling motivated to walk, may consequently perceive the environment as more suitable for active travel.

Parental characteristics and attitudes will be important in determining their own perceptions of the environment as well as their decisions regarding travel modes. For example, not owning a car is an obvious direct promoter of active travel. Yet even those parents who own a car but do not drive frequently may be more active in their local neighbourhood environments, be more familiar with them, and therefore be more likely to decide they are suitable for active travel. In contrast, those with access to a car may perceive the environment as unsuitable simply because of their lack of awareness. In the same way, a parent who has positive prior attitudes towards active travel will be more likely to choose an active travel mode for their children. Research to date has often failed to consider the potentially complex role parents' decision making processes play in controlling their children's travel behaviours and how environmental characteristics interact with these processes. We believe that future research should focus on these roles. The combination of quantitative and qualitative methods may be the best approach to understand this complex process [58].

The framework applies to youth across the age range. However, age will often affect the strength and direction of associations because many physical environmental factors are age specific. As a result, age is an important moderator of children's active travel behaviour. For example, personal and road safety may be more important in determining active transport in children whilst adolescents may have less concern about personal safety; a result of greater freedom and less reliance upon parents. Facilities or destinations to visit may also be important for adolescents, as they seek greater independent mobility. Nevertheless, it is likely that some factors, such as the role of social interactions, will cut across all ages, in this example being important for play in children and companionship in adolescents. There is also evidence to suggest associations between the environment and active travel differ according to gender. For example, Carver et al. [25] found that girls who reported many friends in their neighbourhood, and that their neighbourhood was safe, were more likely to walk for transport. Timperio et al. [34] also noted differential relationships associated with gender, finding that older girls, but not boys, who reported no parks near where they lived were less likely to walk or cycle.

The distance required to travel is likely to also be an overarching moderator of the association between the environment and activity. Regardless of how supportive an environment is for active travel, children may be unlikely to walk or cycle if the distance is too large and the time taken deemed too long. Research has suggested that both children and adolescents are much more likely to walk or cycle to school if the distance is short. Because of the importance of this moderator, it is surprising that so few studies have examined the associations between the environment and travel behaviour according to the distance required to travel. Although many have included distance as a covariate of interest, only one has stratified their analysis by the measure [29]. Future research needs to consider interactions with distance more carefully.

The framework highlights external factors which may influence travel mode decisions but which are external to the neighbourhood and family. They include the weather and climate [30,59], costs of travel and government transport policy [60]. For example, the rising cost of travel, a result of increased fuel prices, may force drivers to consider using their vehicles less frequently. Weather, including warm, dry, cold and wet conditions may also influence walking and cycling behaviour, and government policy which integrates walking and cycling into town planning and transport policies, would be supportive of active transport. These issues are represented in the literature but their detailed consideration does fall within the remit of this review.

## Discussion

The conceptual framework presented here reviews the findings to date in the literature around the environmental determinants of active travel and also highlights areas where future effort should be directed. Environmental factors which are inconsistently associated with active travel behaviours in children warrant greater research. These include provision of facilities, level of urbanisation, route directness, and steepness. School support, in the form of policy, facilities and staff has not been examined and the combined effect of a supportive home, route or destination environment (whether this is school or elsewhere) has not been identified. Further work should also seek account for the main moderators of effects so associations can be confirmed. Indeed, it is likely that many of these environmental variables which show inconsistent associations are being moderated by unmeasured characteristics of the children and parents, such as household socio-economic circumstances. It may also be the case that the importance of environmental features is amplified when they occur together. For example, certain characteristics, such as the availability of parks and greenspaces, may only act as determinants of active travel if they are present on routes that children are likely to take. The possibility of such interactions requires further investigation.

Many of the studies reviewed here used self-reported measures of active travel or computer derived travel paths. Self-reported measures of active travel may be subject to inaccuracies in reporting time taken and distance travelled, although they do allow specific behaviours to be examined. Computer mapping techniques to estimate routes may also introduce inaccuracies because derived routes may not represent actual paths taken [61]. Route choice is an important factor in a decision to use active transport, and whilst the diverse range of methodologies that have been employed in the studies reviewed may be considered a strength, there are problems in determining whether inconsistent findings are associated with real world factors or different study designs. This would be helped by more consistency in future approaches.

Much of the research on this topic has been undertaken in the United States and Australia. More work is required to investigate whether such associations are present in different settings, such as Europe or Asia, countries where the nature of built environments, and the ways in which they are used are quite different. In addition, existing studies have often examined the influence of the environment in small compact geographical areas, often in cities or metropolitan areas. This is often a result of data availability. However, to maximise study power, it is essential that there is significant variation in environments that participants experience [19]. Gathering large data sets for varied environments may be more time consuming and expensive, however such efforts are required. Rural areas offer an environment suitable for comparison with the findings from studies in cities. Previous research has shown that environmental correlates of physical activity differ between urban and rural areas [62,63] yet how this may be associated with active travel is not known.

## Conclusion

In this review we provide evidence of relationships between active travel behaviours and characteristics of the physical environment. Environmental factors which appear to promote active travel in children include safety, social interactions, and the presence of facilities to assist walking and cycling. We provide a conceptual framework that integrates the environment into the wider decision making process around travel choices for children. It is hoped that this will stimulate further research, and also act as a guide for interventions undertaken with the aim of encouraging active travel behaviours.

## Competing interests

The authors declare that they have no competing interests.

## Authors' contributions

JP and APJ conceived of the study and wrote the manuscript. JP conducted the review and synthesised the findings. EVS participated in the study design and coordination and helped to draft the manuscript. All authors read and approved the final manuscript.
